# Functional Foods in Health Promotion and Disease Prevention: Innovations, Evidence and Challenges

**DOI:** 10.3390/foods15040764

**Published:** 2026-02-19

**Authors:** Zheng Feei Ma, Shuchang Liu, Caili Fu, Shaobo Zhou, Yeong Yeh Lee

**Affiliations:** 1 Centre for Public Health and Wellbeing, School of Health and Social Wellbeing, College of Health, Science and Society, University of the West of England, Bristol BS16 1QY, UK; 2 School of Health and Sport Sciences, Liverpool Hope University, Liverpool L16 9JD, UK; 3 Jiangsu Co-Innovation Center of Efficient Processing and Utilization of Forest Resources, College of Chemical Engineering, Nanjing Forestry University, Nanjing 210037, China; 4 School of Science, Faculty of Engineering and Science, University of Greenwich, Chatham ME4 4TB, UK; s.zhou@greenwich.ac.uk; 5 School of Medical Sciences, Universiti Sains Malaysia, Kota Bharu 15200, Malaysia

**Keywords:** functional foods, novel food products, non-communicable diseases, health promotion, disease prevention

## Abstract

Functional foods have attracted increasing scientific and commercial interest due to their potential roles in health promotion and the prevention of non-communicable diseases such as diabetes and cardiovascular diseases. In this review, we will critically examine the current evidence on functional foods by focusing on their classification, bioactive components, biological mechanisms, consumer acceptance and regulatory frameworks. Bioactive compounds, such as polyphenols, dietary fibre and probiotics, from both plant- and animal-origin functional foods, have also been examined in this review. Despite substantial experimental and epidemiological evidence, the translation of functional foods into consistent health benefits remains challenged by variability in bioavailability, food matrix effects, processing conditions and interindividual differences in genetics and gut microbiota. Key mechanistic determinants of bioefficacy, including intestinal transport processes, molecular structure, stereochemistry, and food–drug interactions, are discussed. Consumers’ perception and purchasing behaviour are examined, identifying the influence of product format, socio-demographic characteristics, information sources, health motivation and price sensitivity. Our review also compares the regulatory approaches in the United States, European Union, Japan and China, highlighting the heterogeneity in definitions and health claim substantiation requirements. Finally, emerging opportunities such as metabolic profiling technologies and personalised nutrition are highlighted as future directions to support evidence-based, effective and equitable functional food development.

## 1. Introduction

Functional foods have become increasingly popular globally and are known as “designer foods” and “nutraceuticals” [1]. The global functional food market has experienced remarkable growth and is projected to reach USD 595.49 billion by 2033 from USD 359.81 billion in 2025 [2]. Although the rapid commercial expansion reflects the increasing consumer demands for functional food products, this also raises questions about the robustness of the scientific evidence-based claims that support functional food products [3]. In 1991, the Ministry of Health, Labour and Welfare in Japan introduced the concept of the Foods for Specified Health Uses (FOSHU) system to identify food products with proven health benefits [4]. This pioneering approach later influenced the development in North America and other regions [1].

There are several definitions of functional foods which depend on different regional and regulatory contexts [5,6]. However, in broad terms, they can be explained as foods that provide health benefits beyond basic nutrition [4,5]. The Food and Agriculture Organization (FAO) of the United Nations (UN) has generally defined functional foods with these definitions [7]. According to the FAO of the UN, functional foods are defined as foods that contain, in addition to nutrients, other components that may be beneficial to health [7]. In 1994, the term “functional food” was defined by the Institute of Medicine (IOM) of the U.S. National Academy of Sciences’ Food and Nutrition Board as “any modified food or food ingredient that may provide a health benefit beyond the traditional nutrients it contains” [8]. Under this definition, the scope of the functional food concept had been broadened. For example, foods could be purposefully modified to enhance their health-promoting properties. However, it has also been critiqued for its lack of specificity. It does not clearly distinguish between naturally nutrient-rich foods and those foods that are engineered to deliver targeted physiological effects.

Between 1995 and 1998, a consensus on the definition of functional foods had been reached by more than 100 experts in nutrition and related sciences as part of the European Commission’s Concerted Action on Functional Food Science, which was coordinated by the International Life Sciences Institute. In the European Consensus Document, it is stated that “foods can be regarded as functional if they satisfactorily demonstrate to affect beneficially one or more target functions in the body, beyond adequate nutritional effects, in a way that is relevant to either improved stage of health and well-being and/or reduction in risk of disease” [9]. Although the Academy of Nutrition and Dietetics does not provide a specific definition of functional foods, the Academy of Nutrition and Dietetics includes most healthy food products as functional foods [10]: for example, beans, whole grains, nut, fish, enriched and fortified foods [10]. However, this interpretation of functional foods seems to be very broad. Therefore, there have been some problems associated with these definitions [7,10].

In 2005, a clearer and more precise definition of functional foods was proposed by the Institute of Food Technologies, which is as follows: ‘Functional foods are conventional foods to which specific essential nutrients and/or food components are added for a targeted physiological function’ [11,12]. Therefore, functional foods can be seen as novel food products that have been formulated to contain substances or live microorganisms which have a possible health-enhancing property at a safe and sufficiently high concentration [5]. In 2014, the Functional Food Center (FFC) offered a comprehensive definition of functional foods as “natural or processed foods that contain biologically active compounds which, in defined, effective, and non-toxic amounts, provide a clinically proven and documented health benefit utilising specific biomarkers for the prevention, management, or treatment of chronic disease or its symptoms” [13]. This definition is considered one of the most detailed attempts to operationalise the concept of functional foods. It explicitly connects bioactive components, dose–response relationships and clinical evidence verified through validated biomarkers.

Therefore, in this review, we will synthesise the findings from recent studies on the consumption of functional foods for health promotion and disease prevention. In addition, the review will examine the mechanisms through which bioactive components in functional foods exert physiological benefits, evaluate clinical and regulatory evidence supporting health claims, and discuss consumer perceptions and public health implications. Lastly, the review will identify existing research gaps and methodological challenges that need to be addressed to strengthen the evidence base for functional foods.

### 1.1. Search Methodology

To synthesise the current evidence on functional foods in the context of non-communicable diseases and disease prevention, a comprehensive and systematic literature search was performed in 2025 by using the Web of Science, Scopus and PubMed databases. Relevant eligible articles up to 2025 were identified and retrieved using the following predefined keyword combinations: ‘functional foods’, ‘novel food products’, ‘disease prevention’, ‘health’, ‘non-communicable diseases’ and ‘health promotion’. Inclusion criteria comprised peer-reviewed original research articles and review papers published in English that examined the role of functional foods in the prevention and/or management of non-communicable diseases. Publications such as conference abstracts, commentaries, letters and editorials lacking original empirical data were excluded from the review. Following the screening process, >1000 records were initially identified, about 500 studies were screened after the removal of duplicates, and about 80 studies were ultimately included in the final synthesis.

## 2. Classification of Functional Foods

Functional foods can be categorised into three groups, which are: food ingredients, conventionally used foods and modified foods [1]. Food ingredients include the isolated bioactive components that are added to other food products. Examples of food ingredients are dietary fibre, catechins and lycopene [14]. Conventionally used foods are natural or unmodified foods products such as fruits, fish, dairy, grains and vegetables. Modified foods are food products that have been reformulated, enriched or fortified with specific nutrients or bioactive components to improve their health-promoting properties. Some of the common examples of modified food products include functional coffee with medicinal mushroom, bread enriched with folate, beverages fortified with vitamins and food products supplemented with omega-3 fatty acids [15,16].

## 3. Evidence on Health Benefits of Functional Foods

### 3.1. Functional Foods of Plant Origin

A wide range of plant-based foods and their bioactive phytochemicals have been investigated for their potential roles in disease prevention and health promotion (Table 1) [4]. However, despite the abundance of experimental and epidemiological studies, only a limited number of plant-derived components have been substantiated through rigorous clinical trials to demonstrate reproducible health benefits. Even fewer have met the “significant scientific agreement” threshold established by the U.S. FDA as a prerequisite for the authorisation of health claims [17]. Although Table 1 highlights a range of potential health benefits associated with functional food components, these associations are not consistently confirmed by evidence from human intervention studies [18,19,20,21,22,23]. As a result, there is a risk that such summaries may contribute to a “*health halo effect*”, whereby perceived health benefits are over-generalised or overstated without sufficient clinical validation. Currently, only a select group of plant-derived ingredients are eligible to carry FDA-approved health claims, including soluble fibre from psyllium seed husk, soy protein, oat β-glucan soluble fibre, and sterol- and stanol-ester-fortified margarines [18].

### 3.2. Functional Foods of Animal Origin

Among bioactive compounds derived from animal products, the omega-3 (n-3) polyunsaturated fatty acids have received the greatest scientific attention. Omega-3 is primarily found in fatty fish such as mackerel, herring, salmon, sardines and tuna [1]. The two principal omega-3 fatty acids are docosahexaenoic acid (DHA; 22:6n-3) and eicosapentaenoic acid (EPA; 20:5n-3) [4]. DHA is an integral constituent of cellular membrane phospholipids, particularly in the brain and retina, where it plays a crucial role in maintaining membrane fluidity, signal transduction, and overall cellular function [19]. However, it is important to note that the evidence regarding the health effects of omega-3 fatty acids remains conflicting, as reported by large-scale randomised controlled trials such as the Statin Residual Risk with Epanova in High Cardiovascular Risk Patients with Hypertriglyceridemia trial (STRENGTH) and the Reduction of Cardiovascular Events with Icosapent Ethyl–Intervention Trial (REDUCE-IT) [19]. The STRENGTH trial of 13,078 patients with a median follow-up period of 42 months reported no benefits (primary endpoints including cardiovascular death) (12.0% in intervention group compared to 12.2% in the control group) (hazard ratio of 0.99 (0.90, 1.09), *p* = 0.84), using a combined formulation of EPA and DHA [19]. On the other hand, the REDUCE-IT trial of 8179 patients demonstrated a significant reduction in major adverse cardiovascular events with high-dose purified EPA after a median follow-up period of 4.9 years (4.3% of patients in the intervention group compared to 5.2% in the placebo group) (hazard ratio of 0.80 (0.66, 0.98), *p* = 0.03) [19]. It is suggested that these discrepancies may be attributed to the difference in the dosage and composition of omega-3 used, the biological effects of DHA versus EPA and the choice of comparator oils. Therefore, these studies underscore the need for cautious interpretation of functional food health claims, including those about omega-3. Also, a more nuanced, evidence-based approach should be employed to prioritise high-quality clinical data, rather than data extrapolation from preclinical, animal and small-scale human studies.

Another major class of biologically active animal-derived components that has attracted increasing scientific and commercial interest is probiotics. Probiotics are defined as “viable microorganisms that confer health benefits to the host when administered in adequate amounts” [1]. While *Lactobacillus acidophilus* remains one of the most studied species, several other strains such as *L. johnsonii La1, L. reuteri, L. rhamnosus GG,* and *L. casei Shirota* are widely incorporated into functional food products [20]. However, much of the supporting evidence is derived from animal and small-scale human studies. There is a lack of large-scale clinical trials with extended follow-up on participants and standardised primary outcome measures to allow for comparisons between different studies investigating the effect of probiotic consumption [20]. In addition, strain-specific effects are not always well characterised. Moreover, the survivability of probiotic strains through food processing, storage and gastrointestinal transit remains an important determinant of efficacy [21]. Consequently, while the mechanistic rationale for probiotics is biologically plausible, inconsistencies in clinical outcomes and regulatory ambiguity continue to limit definitive conclusions about their role in disease prevention and management [4,22].

## 4. Bioavailability and Efficacy of Functional Foods

Functional foods are characterised by highly complex food matrices that contain a diverse range of bioactive components, including polyphenols, vitamins, minerals and other phytochemical compounds [23]. These bioactive compounds collectively contribute to the biological activities and health-promoting effects of functional foods through their antioxidant, anti-inflammatory and metabolic-regulating properties [24]. Therefore, the bioavailability and efficacy of bioactive compounds in functional foods represent one of the major determinants of these health-promoting properties. Bioavailability is one of the important steps to ensure the bioefficacy of bioactive food compounds in functional foods [25]. A wide range of factors influence the bioavailability of bioactive food compounds, including bioaccessibility, the food matrix effect, membrane transporters, molecular structure, and the activity of metabolising enzymes [26]. Enhancing bioavailability is essential for improving bioefficacy, as greater absorption and metabolic utilisation increase the likelihood that bioactive components exert measurable physiological effects.

Due to the complex chemical nature of food bioactive compounds and the distinct absorption mechanisms of hydrophilic and lipophilic molecules, there are some challenges that remain to ensuring that these bioactive compounds remain stable, absorbable and physiologically effective once consumed [27]. Some bioactive compounds including polyphenols, carotenoids, lutein and omega-3 fatty acids are chemically unstable and can degrade during processing, storage or digestion, which may limit their functional potential [28].

The rate and extent of absorption of bioactive food compounds can vary considerably between individuals [29]. This interindividual variability in bioavailability is influenced by several key factors, including dietary composition, genetic background, and the composition and metabolic activity of the gut microbiota [30]. Such variability can profoundly affect the physiological responses to functional foods and their bioactive constituents. For example, polyphenols are generally poorly absorbed, with reported absorption rates ranging from 0.3% to 43%, and their circulating plasma metabolite concentrations are often low [31]. These observations highlight the complexity of linking dietary intake to biological effects and underscore the importance of considering personalised and microbiome-mediated factors when evaluating the efficacy of functional foods.

## 5. Factors Affecting Bioavailability

The bioavailability of bioactive compounds in functional foods can be influenced by a wide range of environmental and biological factors (Table 2). For example, gut microbiota composition, genetic polymorphisms and food processing techniques are used [32]. Also, the conversion of polyphenols and isoflavones into bioactive metabolites can be affected by the interindividual variability in the gut microbial metabolism, which may partly explain the inconsistent physiological outcomes across different studies [30]. In addition, the nutrient bioaccessibility can be either enhanced or reduced by the thermal or mechanical food processing used, which depends on the food matrix and compounds in the functional foods [24].

### 5.1. Transport Mechanisms

The transport mechanisms operating within the intestinal lumen are among the most critical factors influencing the bioavailability of both ingested food bioactive compounds [33]. These mechanisms include passive diffusion, facilitated diffusion, and active transport. Since the majority of the bioactive food compounds lack the optimal physicochemical properties required for passive diffusion, transmembrane transporters play an essential role in mediating permeability [34]. Two major classes of transporters regulate this process: uptake transporters and efflux transporters. The uptake is facilitated by transport proteins such as vitamin transporters, the glucose transporter (GLUT) family, the sodium–glucose (SGLT) cotransporter family and the organic anion transporter (OAT1), which enhance compound absorption across the intestinal barrier [25]. Conversely, the ATP-binding cassette (ABC) family of transporters including P-glycoprotein (P-gp) and the breast cancer resistance protein (BCRP) mediate efflux mechanisms that can restrict the absorption and systemic availability of both pharmaceuticals and bioactive food constituents [35].

Competition for transport via organic anion transporters (OATs) may influence the disposition of bioactive food compounds and drugs [36]. In cell culture models, such competition may result in the retention of certain drugs within cells, potentially leading to prolonged plasma half-lives and altered pharmacokinetics [37]. A study by Whitley et al. reported that their study demonstrated a high binding affinity of ellagic acid (a polyphenolic compound found in fruits and nuts), particularly for human OAT1 (hOAT1) [38]. Their findings suggested potential transport-mediated interactions between ellagic acid and several classes of drugs, including β-lactam antibiotics, angiotensin-converting enzyme inhibitors and nonsteroidal anti-inflammatory drugs (NSAIDs). Such interactions highlight the possibility that dietary polyphenols may modulate drug absorption, distribution, and elimination, underscoring the importance of understanding food–drug transporter interactions in both nutrition science and pharmacology [39].

Some transport proteins such as plasma membrane fatty acid-binding protein (FABPpm), fatty acid transport protein 4 (FATP4) and CD36 have been associated with the transport of lipids across the intestinal barrier [40]. Therefore, it is suggested that when multiple food nutrients share the same family of membrane transporters, competitive inhibition may occur, thereby reducing the absorption efficiency of certain compounds. A study by Richelle et al. reported that the consumption of plant sterols decreased the intestinal absorption of cholesterol, carotenoids and α-tocopherol in normocholesterolaemic subjects, which is most likely through competition for shared lipid transporters [41]. Their study findings suggest that interactions among dietary lipophilic compounds can significantly influence nutrient bioavailability, underscoring the complexity of absorption processes and the need to consider nutrient–nutrient competition in the design of functional foods and nutritional interventions [42].

### 5.2. Structure of Bioactive Compounds

The molecular structure of a bioactive compound has been identified as one of substantial influences on its intestinal absorption and bioavailability [43]. For example, bioactive compounds with a high molecular weight such as oligomeric proanthocyanidins and complex lipids are generally unable to cross the intestinal epithelial barrier unless they are first hydrolysed and degraded into smaller units [44]. Similarly, the sugar moiety of flavonoids plays an important role in determining their absorption efficiency in humans [45]. As one of the most prevalent forms found in nature, flavonoids conjugated with β-glucosides are absorbed only to a limited extent in their intact form [46]. They are typically hydrolysed by intestinal enzymes, such as β-glucosidases and lactase-phlorizin hydrolase, within the small intestine. In contrast, flavonoids containing an additional rhamnose moiety, such as quercetin glycosides from tea, must reach the large intestine. This is where their sugar residues are cleaved by the gut microbiota to enable subsequent absorption of the aglycone form [47]. These structural distinctions highlight how molecular complexity and glycosylation patterns significantly shape the absorption kinetics, metabolic fate, and ultimately the bioefficacy of dietary bioactive compounds [48].

Another important factor that determines the absorption, metabolism and bioefficacy of bioactive compounds is their isomeric configuration [49]. Flavonoids and other phytochemical compounds with different stereochemical configurations often exhibit markedly distinct bioavailability profiles [25]. Variations in the absorption and metabolic fate have been reported between cis- and all-trans-lycopene isomers, between (–)-epicatechin and (+)-catechin, as well as for the biological activities of the (R/S) isomers of equol [50,51,52,53]. For example, when using the plasma and urinary analyses, an R:S ratio of 39:69 for hesperetin-7-glucoside was reported, which suggested that the S-enantiomer may be more readily absorbed and bioavailable in humans [51]. Similarly, approximately 95% of lycopene, which is a carotenoid abundant in tomatoes, exists in the all-trans form in raw tomatoes. However, in human plasma, cis-isomers account for nearly 50% of the total lycopene, likely resulting from isomerisation during digestion and the greater intestinal permeability of cis-configured molecules [54]. These examples underscore that molecular stereochemistry, including cis/trans and enantiomeric configurations, significantly influences not only the bioavailability but also the biological potency of food bioactive compounds.

### 5.3. Interactions Between Bioactive Compounds and Drugs

Once a drug or bioactive food compound enters the enterocyte, it may undergo metabolic transformation mediated by cytochrome P450 (CYP) enzymes (Phase I metabolism) [25]. During this phase, these compounds will be modified to xenobiotic structures through oxidation, reduction, or hydrolysis reactions. However, polyphenols generally do not undergo Phase I. Instead, they primarily undergo Phase II metabolism, which involves the conjugation reactions catalysed by enzymes such as catechol-O-methyltransferases (COMT) for methylation, sulphotransferases (SULT) for sulphation, and uridine-5′-diphosphate glucuronosyltransferases (UDPGT) for glucuronidation [55]. These conjugation processes result in metabolites that differ structurally and functionally from the original dietary compounds [46].

The activity of CYP enzymes can be inhibited or induced by co-administered bioactive compounds or drugs, thereby altering the systemic bioavailability of other compounds [56]. For example, the lignan sesamin (in sesame seeds), has been reported to significantly increase γ-tocopherol levels in humans through inhibition of CYP activity [57]. Another example of food–drug interaction involves grapefruit juice, which contains bergamottin, a furanocoumarin compound implicated in CYP3A4 inhibition [58]. Co-administration of grapefruit juice with CYP3A4 substrates can significantly elevate oral drug bioavailability, leading to supratherapeutic plasma concentrations and potential toxicity [59].

## 6. Consumer Perception, Acceptance and Public Health Implications

Chronic diseases including cancer, diabetes and cardiovascular disease remain the leading causes of morbidity and mortality worldwide [60]. In addition, they represent major public health concerns in populations. These chronic diseases are strongly associated with unhealthy dietary patterns, which are characterised by excessive consumption of refined sugar, salt, cholesterol and saturated fats [61]. In response, there has been increasing societal awareness of the role of diet in the prevention of chronic disease and a significant increase in consumer demand for healthier food choices. As a result, the development of functional foods has gained growing scientific and commercial interest in the prevention of chronic disease [62,63]. Table 3 shows some of the key factors that can influence consumers’ perception of and purchasing behaviour towards functional foods.

### 6.1. The Combination of Food Ingredients and Carriers

One of the factors that have been reported to influence the consumer acceptance of functional foods is the combinations of ingredients and carriers [6]. A study by Ares et al. reported that when yoghurts were added with functional ingredients such as antioxidants and fibre, they were more likely to be chosen by consumers [63]. In addition, the authors reported that consumers were more likely to be interested in purchasing yoghurts enriched with fibre than regular yoghurts. In a study by Van Kleef et al., participants reported that they had higher preferences for healthier carriers such as yoghurt and margarine when compared with indulgence-type foods including ice-cream, chocolate and chewing gum [64]. Also, a study by Verbeke et al. reported that fibre-enriched cereals were more readily accepted by consumers than calcium-fortified juice [65]. This is largely because the calcium-fortified juice was perceived as a less natural or less appropriate food–nutrient combination. Similarly, Bech-Larsen and Grunert reported that consumers tend to view naturally wholesome foods such as yoghurt as more suitable and credible carriers for functional ingredients than unwholesome food products such as spreads [66]. Another study of German consumers by Wortmann et al. reported that selenium-rich apples were more likely to be purchased than non-fortified ones, if it was advertised with approved nutrition and health claims [67]. In mainland China, a study by Huang et al. reported that the perceived attractiveness and purchase intention toward functional foods were influenced more strongly by the type of carrier product than by the specific health benefits claimed [68].

### 6.2. Socio-Demographic Characteristics

Socio-demographic characteristics play an important role in shaping the consumer acceptance and purchasing behaviour toward functional foods [6]. Evidence from studies suggests that key determinants include age, gender, household composition, educational attainment, geographical location, marital status and nationality [6,68,69]. A study by Huang et al. in mainland China reported that women with a middle level of education appeared to be the primary target consumers for functional foods aimed at reducing body fat [68]. On the other hand, older adults with a similar middle level of educational backgrounds and upper-middle income levels constitute the main market segment for functional food products that are designed to lower the risk of osteoporosis and cardiovascular disease [68]. These findings suggest that functional foods addressing general health and wellbeing may appeal to a broad consumer base, while those formulated for the prevention or management of specific health conditions should be strategically tailored to particular demographic groups. Such socio-demographic variations highlight the importance of targeted marketing and culturally sensitive communication strategies in promoting functional food adoption across diverse populations [70]. This underscores the need for functional food market segmentation and targeted communication strategies in functional food development and marketing, ensuring that health claims resonate with the needs, motivations, and capacities of distinct consumer populations.

In addition, several studies reported that women and older consumers have greater interest in functional foods than other demographic groups [68,71,72,73]. It is possible that women tend to value not only the general health benefits of functional foods but also their potential to enhance physical appearance and personal image. For older consumers, they are primarily motivated by food products that strengthen their body’s natural defences and prevent age-related diseases. A study by Huang et al. found that urban consumers have stronger preferences for functional foods than rural consumers, although this advantage diminishes when the attractiveness carrier products decrease [68]. For yoghurt and non-alcoholic beverages, urban residents accounted for >74% of enthusiastic supporters, with statistically significant differences across clusters (*p* < 0.05) [68]. This pattern may reflect both the greater accessibility and affordability of functional foods in urban markets and the information overload urban consumers face, which can lead to more cautious or sceptical purchasing decisions when confronted with less appealing products. Huang et al. reported that the influence of marital status on the Chinese consumer acceptance of functional food appears to be less consistent, which suggests that it may not be a major determinant of functional food acceptance.

### 6.3. Information Channels

The production of functional foods often involves the use of novel food technologies such as the addition of new or modified bioactive ingredients into food products [74]. Although these technological processes are scientifically justified, they may appear unfamiliar to consumers, which may lead to scepticism and reluctance toward adoption [6]. Moreover, some consumers may face difficulty verifying immediate and tangible health effects at the point of consumption. As these health benefits are often long-term and invisible, it is possible that the consumer purchase decisions rely heavily on perceived credibility, rather than direct experience. Consequently, the degree of consumer trust which is shaped by perceptions of science, regulation, and brand integrity plays a central role in determining the consumer acceptance of functional foods [6].

A study by Huang et al. reported that perceived trust in friends’ recommendations had emerged as the most significant factor in shaping the consumer acceptance of functional foods [68]. The authors reported that the Chinese consumers are more likely to rely heavily on interpersonal trust and recommendations from acquaintances than by impersonal marketing sources. Such reliance reflects a belief that advice from trusted social networks helps to identify some credible functional food products that align with personal needs and values [75]. Given the low public confidence in China’s food industry, this pattern is particularly salient, which has been undermined by repeated food safety scandals [76,77,78]. Therefore, peer recommendations and functional food product trials are perceived as more authentic and reliable sources of information than mass media or advertising for the consumers.

### 6.4. Consumer Motivations

Health motivation has been identified as one of the important psychological drivers that influence the consumers’ acceptance and consumption of functional foods [6,79]. It is defined as “consumers’ goal-directed arousal to engage in preventive health behaviours”. Several studies have reported that consumers with stronger health motivations such as maintaining overall wellbeing or preventing disease are more likely to develop positive attitudes and purchase intentions toward functional foods [6,79,80,81]. Chinese consumers who placed greater importance on their mobility health such as optimal functioning of joint, muscles and bones reported a higher willingness to purchase functional foods [79]. In addition, in a study by Chang et al., consumers who were more health-conscious, valued healthy lifestyles and actively engaged in health-promoting dietary behaviours had a greater willingness to purchase functional beverages [81]. Collectively, these findings suggest that functional food consumption is largely goal-driven, with health-motivated consumers perceiving such products as proactive strategies for disease prevention and long-term wellbeing.

### 6.5. Price of Functional Food Products

Several studies have reported that the price of functional foods is one of the key determinants of the consumers’ acceptance and purchase intention [63,79,82,83]. Although consumers are willing to pay a reasonable premium for functional food products, the influence of price on consumer behaviour appears to operate in two opposing directions. On one hand, higher prices of functional food products may discourage purchase due to budget constraints or scepticism about value for money. On the other hand, a higher price may enhance perceived product quality or efficacy, particularly when consumers associate price with trustworthiness or superior formulation [63].

A systematic review of peer-reviewed published studies to examine whether consumers are willing to pay a price premium for healthier foods by Alsubhi et al. reported that of the 26 experiments, consumers in 23 experiments (88.5%) were willing to pay between 5.6% and 91.5% more for healthier food products, with a mean premium of 30.7% [84]. Consistently, consumers demonstrated a positive willingness to pay for foods reduced in fat or enriched with whole grains, fruits and vegetables. On the other hand, the willingness to pay for food products that were low in salt or combined multiple nutrient reductions (e.g., low fat and low sugar) produced more mixed results. The authors also reported that older adults (aged ≥60 years), women, individuals living with obesity and those actively pursuing a healthy lifestyle were more likely to pay higher prices for healthier food products. In contrast, younger consumers, those with a healthy weight and highly educated consumers tended to be less willing to pay a price premium.

A study investigating the consumer choice of functional and regular yoghurts by Ares et al. reported that price had a relative importance of ~20% in influencing consumer purchase and consumption of functional foods [63]. Also, the authors reported that consumers motivated by specific health concerns were less price-sensitive, suggesting that health orientation moderates the effect of price. Similarly, another study in the Western Province of Sri Lanka by Narayana et al. reported that about one-third of participants (32%) prioritised price considerations over other factors such as taste and health benefits [85]. In a study investigating the antecedents of functional food purchases in Chinese consumers, Huang et al. reported that the negative influence of price can be offset by high health consciousness, as consumers with greater concern for personal wellbeing are more willing to pay a premium for health-related benefits [68].

However, the consumers’ willingness to pay has some clear limits. For example, a study using a mixed methodological approach by Mirosa and Mangan-Walker reported that Chinese consumers were generally unwilling to pay more than 40% above the standard price [79]. On the other hand, Menrad reported that European consumers would pay only 30–50% more for functional foods [86]. Therefore, these study findings suggest that although health-related motivation can increase tolerance for higher prices, the premium consumers are willing to pay remains bounded, highlighting the need for pricing strategies that balance perceived value, affordability, and accessibility in different markets.

## 7. Methodological and Regulatory Challenges

### 7.1. The United States (U.S.)

In the U.S., no formal definition has been provided by the Federal Food, Drug, and Cosmetic Act (FFDCA). As a result, the Food and Drug Administration (FDS) does not have a dedicated regulatory framework for the rapidly expanding functional food market [87]. Therefore, functional food products are assessed under the existing regulatory framework, which depends on their food composition and intended health claims. This fragmented approach also creates regulatory ambiguity, which allows for flexibility in the marketing of functional food products [87]. Consequently, there is increasing concern over the adequacy of the current safeguards for ensuring the scientific validity and safety of the health claims for the functional food products made by the functional food manufacturers [87]. The introduction of food labels by the Nutrition Labelling and Education Act (NLEA) developed in 1990 described the relationship between a nutrient and health-related condition [88]. These potential health benefit claims for functional foods should be approved by the FDA before they are placed on the market.

In 1994, the implementation of the Dietary Supplement Health and Education Act (DSHEA) classified food additives as amino acids, vitamins and minerals used to improve health conditions [1]. In addition, the introduction of the Significant Scientific Agreement (SSA) requires the health claims to be supported by credible science evidence that demonstrates both safety and efficacy in humans [89]. This concept also emphasises the need to document the biological activity, mechanism of actions and potential pharmacological effects of the functional components. The FDA must be notified by the food companies within 30 days of marketing when they are making the structure and function claims on their food labels [89]. While the implementation of the FDA Modernization Act (FDAMA) in 1997 smoothened the FDA preapproval process by implementing the authoritative statements on food labels as health claims, the FDA should be notified by food manufacturers who intend to use such authoritative statements 120 days before the functional food marketing. The authoritative statements should be derived from the credible scientific consensus within U.S. government bodies such as the National Institutes of Health (NIH) (Table 4) [1].

### 7.2. European Union

In the European Union (EU), the use of health claims for functional food products is strictly regulated under the Regulation (EC) No. 1924/2006, which came into force in 2007 [90]. The regulation has established a unified framework to govern the wording, substantiation and communication of nutrition and health claims across all EU member states. The regulation is designed to harmonise regulatory standards, protect consumers from misleading marketing, and ensure transparency through the EU register of permitted health claims [91]. Under the regulation, all health claims must undergo scientific evaluation and approval by the European Food Safety Authority (EFSA) before being authorised for use [91].

### 7.3. Japan

In Japan, functional foods are regulated under two official systems, which are the FOSHU system and the Foods with Function Claims (FFC) framework [91]. The FOSHU system requires food manufacturers to provide scientific evidence for both the efficacy and safety of the functional food products [92]. Functional food products undergo a rigorous assessment before they are permitted to display the approved health claims. In 2015, the FFC framework was introduced to streamline market access. Under the FFC framework, food companies can self-substantiate their health claims by using the scientific evidence derived from systematic reviews or clinical trials [93]. In addition, prior to the marketing activities, food companies need to notify the Consumer Affairs Agency (CAA) without requiring formal pre-approval [91]. While the FFC system has significantly expanded the number of functional food products entering the functional food market, it has also raised some concerns, especially about the variable scientific quality, transparency and post-market surveillance. In addition, the reliance on self-submission by food companies may compromise consumer confidence and blur the distinction between verified and unverified health claims [94,95].

### 7.4. China

In China, the National Medical Products Administration (NMPA) regulates functional foods, which are classified as “health foods” [96]. To obtain market approval, food manufacturers must follow one of two regulatory pathways, which are registration or notification. For example, the registration route applies to food products containing novel ingredients or those imported from abroad [97]. Therefore, these food products require a comprehensive evaluation of safety, efficacy and product formulation. In contrast, the notification system is reserved for food products that use pre-approved ingredients listed in the national catalogue. The health functions that can be claimed for the functional food products are limited to a government-approved list of authorised functions [91].

From a scientific perspective, the comparison of different regulatory frameworks across the different regions such as the US, EU, Japan and China highlights that there is a need for a more standardised and rigorous approach to the evidence generation in functional food research. On the other hand, from an industry perspective, these divergent regulatory frameworks and standards have created both opportunities and challenges for the development and commercialisation of functional food products. For example, in the EU and Japan, the flexible framework may help to facilitate the innovation and rapid market entry but concerns may be raised regarding the credibility and consistency of health claims about functional food products. In contrast, the stricter systems in the EU and China may enhance the consumer protection but constrain functional food product development and increase these functional food development costs. It is suggested that greater transparency and making these standards and frameworks international could further support a more responsible innovation and promote the sustainable growth of the functional food industry.

## 8. Summary and Future Directions

The functional food market is rapidly expanding, which is not just a momentary trend, but also reflects the shift in society’s attitudes toward health and personalised nutrition. However, translating the potential of functional foods into robust and reproducible health benefits remains challenging. The traditional methods used to discover functional food ingredients such as antioxidants are time-consuming. However, the introduction of AI technology to the functional food industry is a strategic response to the current methodological challenges. The advances in big data and AI enable the analysis and interpretation of vast datasets, including metabolomics and genomics, for uncovering the potential health benefits of the bioactive compounds in functional food products. In addition, machine learning methods can be used to identify the optimal processing conditions of functional foods by reducing the loss of antioxidants, flavonoids and bioactive compounds. Therefore, by determining the optimised processing routes that retain the bioactive compounds of interest in the functional foods, the functional and nutritional profiles of the functional foods can be improved. Furthermore, the application of AI in precise nutrition can help to predict how the functional ingredients may affect the health and wellbeing status of individual consumers. One example is that by using these machine learning models, the individual consumer’s genetic background can be analysed and their response to a functional ingredient can then be predicted. The predictive power of these machine learning models will then further contribute to the development of precise nutrition as a tool to prevent chronic diseases.

There is growing interest in applying metabolic profiling technologies within the food science industry to support the development of personalised functional foods for consumers. Such approaches can inform multiple stages of functional food development, including ingredient selection, optimisation of bioactive composition and evaluation of individual metabolic responses. For example, metabolomics can be employed to identify and quantify the different types of metabolites in functional foods. The application of metabolomics is helpful to facilitate the identification of new functional food ingredients and the development of new functional food products. Advancing these areas will be critical to translating nutraceutical and functional food research into evidence-based, effective and equitable dietary interventions. However, there are still some challenges associated with this. For example, the roles, possible side effects, safe consumption ranges and changes in these functional food ingredients in the body after digestion remain to be investigated. Therefore, future well-designed randomised controlled trials of functional food consumption are required to establish the optimal dosages, clarify the long-term safety and elucidate the potential interactions between different bioactive components of functional foods

Future studies should also address challenges related to functional food product standardisation and regulatory frameworks. Regulatory requirements vary considerably across regions and countries, making it difficult to define consistent criteria and product categories for functional foods. This variability complicates the demonstration of the bioavailability and efficacy of bioactive compounds required to substantiate health claims and support the responsible marketing of functional food products.

## Figures and Tables

**Table 1:** Some common examples of functional foods of plant and animal origin, key bioactive components, and associated health benefits.

Functional Foods	Some Key Bioactive Components	Proposed Mechanisms of Actions	Health Benefits	References
*a) Plant origin*				
Fruits (e.g., berries, goji, citrus)	Flavonoids, polyphenols and vitamin C	Antioxidant and anti-inflammatory effects; endothelial function	Reduced oxidative stress; improved vascular health; cognitive protection	[14]
Legumes (e.g., lentils, soybean)	Isoflavones, plant protein and fibre	Oestrogen-like activity; cholesterol reduction; gut microbiota modulation	Improved lipid profiles; bone health; cardiometabolic protection	[14]
Nuts and seeds	Unsaturated fatty acids, phytosterols and tocopherols	Lipid lowering; anti-inflammatory pathways	Improved lipid metabolism; reduced cardiovascular risk	[23]
Tea (e.g., green, black)	Catechins and theaflavins	Antioxidant activity; modulation of glucose and lipid metabolism	Reduced cardiometabolic risk; cognitive and mental health support	[23]
Functional coffee with mushrooms (e.g., lion’s mane, reishi, chaga, cordyceps)	Chlorogenic acids, caffeine; mushroom β-glucans, triterpenoids, hericenones	Antioxidant and anti-inflammatory effects; neurostimulation; immune modulation; potential gut–brain axis interaction	Improved alertness; potential cognitive support and stress resilience; immune function support	[14,16]
*b) Animal origin*				
Dairy products (e.g., yoghurt, kefir)	Probiotics, bioactive peptides and calcium	Gut microbiota modulation; improved nutrient absorption	Improved gut health; bone health; immune support	[14]
Fatty fish (e.g., salmon, mackerel)	Omega-3 fatty acids (EPA and DHA)	Anti-inflammatory effects; neuronal membrane integrity	Cardiovascular health; cognitive ageing and dementia risk reduction	[18]
Bee products (e.g., propolis, royal jelly)	Flavonoids and bioactive peptides	Immunomodulatory and antioxidant effects	Immune and metabolic health	[18]
Meat and poultry (functional/enriched)	Iron, zinc and vitamin B12	Oxygen transport; immune and neurological function	Reduced micronutrient deficiencies; improved energy metabolism	[18,23]
Goat milk and fermented goat dairy	Medium-chain fatty acids and bioactive peptides	Enhanced fat digestion; anti-inflammatory effects	Gut health; nutrient absorption	[14,20]

**Table 2:** Some common factors that affect the bioavailability and strategies to enhance the efficacy of functional food components.

Category	Challenges	Examples of Bioactive Compounds That May Be Affected	Technological Innovations to Enhance Efficacy	References
Physicochemical stability	Degradation during processing, storage and digestion	Polyphenols, carotenoids and omega-3 fatty acids	Nanoencapsulation; microencapsulation; emulsification; controlled-release coatings	[25]
Food matrix interactions	Binding to macronutrients, limited solubility and poor release	Minerals (e.g., iron, zinc) and flavonoids	Optimising food matrix composition; co-delivery with lipids or emulsifiers	[25,26]
Gastrointestinal conditions	pH variations, enzymatic degradation and poor intestinal permeability	Probiotics, peptides and phytosterols	pH-sensitive delivery systems; enteric coatings; lipid-based carriers	[25]
Genetic and physiological variability	Polymorphisms affecting absorption and metabolism	Folate, isoflavones and polyphenols	Nutrigenomic approaches; personalised nutrition models	[29]
Gut microbiota composition	Microbial metabolism affecting conversion to active metabolites	Polyphenols, flavonoids and isoflavones	Probiotic or prebiotic co-formulations; synbiotic functional foods	[25]
Processing effects	Thermal, mechanical and chemical alterations	Vitamins (e.g., C and E) and phenolic compounds	Cold processing; high-pressure processing; microencapsulation during heat treatment	[28]
Targeted delivery	Low specificity of bioactive delivery to target tissues	Omega-3 fatty acids and curcumin	Smart nanocarriers; bio-responsive hydrogels; lipid nanoparticles	[29]

**Table 3:** Some key factors that can influence the consumer perception and purchasing behaviour towards functional foods.

Factor Category	Specific Factors	Influence on Consumer Perception	Implications for Purchasing Behaviour	References
Perceived health benefits	Disease prevention claims; general health maintenance; energy, immunity, cognitive support	Strong determinant of perceived value and relevance	Increases willingness to try and repurchase, particularly among health-conscious consumers	[6,63]
Taste and sensory attributes	Flavour, texture, aroma and appearance	Determines overall acceptability	Poor sensory quality can outweigh perceived health benefits and deter purchase in consumers	[6]
Naturalness and ingredient transparency	Clean label; minimal processing; recognisable ingredients	Reinforces perceptions of safety and authenticity	Increases preference for “natural” or minimally processed products	[6]
Price and affordability	Cost relative to conventional foods; perceived value for money	Influences accessibility and equity	High price may limit uptake despite positive perceptions	[63]
Knowledge and nutrition literacy	Understanding of functional foods and bioactive components	Reduces confusion and scepticism	Informed consumers more likely to adopt functional foods	[6]

**Table 4:** Regulatory frameworks and key methodological challenges for functional foods across regions.

Region	Regulatory Authority/Framework	Definition & Regulatory Approach	Evidence Requirements for Health Claims	Key Methodological and Regulatory Challenges
The United States (U.S.)	Food and Drug Administration (FDA); Federal Food, Drug, and Cosmetic Act (FFDCA); Nutrition Labeling and Education Act (NLEA); Dietary Supplement Health and Education Act (DSHEA)	No formal legal definition of functional foods. Products are regulated under existing food and dietary supplement frameworks depending on composition and intended claims.	Health claims require prior FDA approval; structure–function claims require notification within 30 days of marketing. Claims should be supported by the Significant Scientific Agreement (SSA), demonstrating safety and efficacy in humans.	Fragmented regulatory framework creates ambiguity and flexibility in marketing. Concerns remain regarding the adequacy of safeguards to ensure scientific validity and consumer protection.
European Union (EU)	European Food Safety Authority (EFSA); Regulation (EC) No. 1924/2006	Unified and harmonised regulatory framework governing nutrition and health claims across all EU member states.	All health claims must undergo scientific evaluation and authorisation by EFSA before use. Only approved claims may be communicated.	High evidentiary threshold limits approval of new claims; many proposed claims fail due to insufficient or inconsistent human evidence.
Japan	Consumer Affairs Agency (CAA); FOSHU and Foods with Function Claims (FFC) systems	Dual system: FOSHU requires pre-approval, while FFC allows self-substantiated claims following notification.	FOSHU: rigorous assessment of safety and efficacy. FFC: claims supported by systematic reviews or clinical trials, without formal pre-approval.	Rapid market expansion under FFC raises concerns over variable scientific quality, transparency and post-market surveillance. Self-substantiation may blur distinctions between verified and unverified claims.
China	National Medical Products Administration (NMPA)	Functional foods classified as “Health Foods” and regulated under registration or notification pathways.	Registration required for products with novel or imported ingredients; notification applies to products using pre-approved ingredients. Health claims restricted to a government-approved list.	Limited scope of permitted health functions; regulatory burden for novel products may constrain innovation despite clearer classification.

## Data Availability

No new data were created or analysed in this study.
